# Supplementary material for: EspP2 Regulates the Adhesion of Glaesserella parasuis via Rap1 Signaling Pathway
Source: Int J Mol Sci. 2024 Apr 22;25(8):4570. doi: 10.3390/ijms25084570 (PMC11050538; doi:10.3390/ijms25084570)
Supplement: Supplementary file 1 [file ijms-25-04570-s001.zip › ijms-2918640-supplementary.pdf]

Supplementary Material

Supplementary Tables

Table S1. Strains and plasmids were used in this study.

Primers	Primer sequences (5′→3′)	products (bp)
P1（*EspP2*L-F）	ctatgacatgattacgaattcCGAAACTTGCGGAACATCTT	620 bp
P2（*EspP2*L-R）	gcagggcttcccaaccttacTATGTTCTCCTTCAAATCATTGG	
P3（*EspP2*R-F）	ggggttcgaaatgaccgaccCATCAATATAGGGTAATTTAAGAA	611 bp
P4（*EspP2*R-R）	caggtcgactctagaggatccATAGACAACGAAAGAGAGTACC	
P5（Kan-F）	GTAAGGTTGGGAAGCCCTGC	935 bp
P6（Kan-R）	GGTCGGTCATTTCGAACCCC	
P7（GPS-F）	ACAACCTGCAAGTACTTATCGGGAT	276 bp
P8（GPS-R）	TAGCCTCCTGTCTGATATTCCCACG	
P9（*EspP2*-F）	TGAAGCTCAGACTTATTGGGCA	2202 bp
P10（*EspP2*-R）	GCCTATACTTAACTTATCAT	
P11（pK18-F）	CTGGCACGACAGGTTTCC	342 bp
P12（pK18-R）	GCCTCTTCGCTATTACGC	
P13（*rEspP2*-F）	CAGACTTATTGGGCAAGTG	2271 bp
P14（*rEspP2*-R）	GAACGAGTATCTTACATTAGAATTA	
P15（32a-*rEspP2*-F）	gccatggctgatatcggatccCAGACTTATTGGGCAAGTG	2313 bp
P16（32a-*rEspP2*-R）	gtggtggtggtggtgctcgagGAACGAGTATCTTACATTAGAATTA	
P17（CL-1-F）	CGGATGGCTGTCATTGGG	263 bp
P18（CL-1-R）	GGCGAAGGTTTTGGATAGG	

P19（OCLN-F）	CCCTTTCGGACTATGCGG	282 bp
P20（OCLN-R）	CCGTCGTGTAGTCTGTCTCG	
P21（Actin-F）	CTTCCTGGGCATGGAGTCC	201 bp
P22（Actin-R）	GGCGCGATGATCTTGATCTTC	
P23（RAP1B-F）	GGCTCAGGAGGCGTTGGAAA	240 bp
P24（RAP1B-R）	TGTGGACTGTGCTGTGATGGAATA	

Table S2. Reads and reference genome comparison list.

Sample name	Total reads	Total mapped	Multiple mapped	Uniquely mapped	Read-1	Read-2
E0_1	43966372	41750819 (94.96%)	1439158 (3.27%)	40311661 (91.69%)	20259617 (46.08%)	20052044 (45.61%)
E0_2	43715322	41579416 (95.11%)	1384224 (3.17%)	40195192 (91.95%)	20180590 (46.16%)	20014602 (45.78%)
E0_3	41046548	39076519 (95.2%)	1314749 (3.2%)	37761770 (92.0%)	18935780 (46.13%)	18825990 (45.86%)
E12_1	40370164	38485663 (95.33%)	1167655 (2.89%)	37318008 (92.44%)	18721221 (46.37%)	18596787 (46.07%)
E12_2	44944598	42858973 (95.36%)	1356855 (3.02%)	41502118 (92.34%)	20839307 (46.37%)	20662811 (45.97%)
E12_3	44151258	42135910 (95.44%)	1382831 (3.13%)	40753079 (92.3%)	20460195 (46.34%)	20292884 (45.96%)
E36_1	42907506	40795362 (95.08%)	1372463 (3.2%)	39422899 (91.88%)	19818134 (46.19%)	19604765 (45.69%)
E36_2	44894094	42710168 (95.14%)	1363724 (3.04%)	41346444 (92.1%)	20761566 (46.25%)	20584878 (45.85%)
E36_3	45841584	43510641 (94.92%)	1410640 (3.08%)	42100001 (91.84%)	21178863 (46.2%)	20921138 (45.64%)

Supplementary Figures

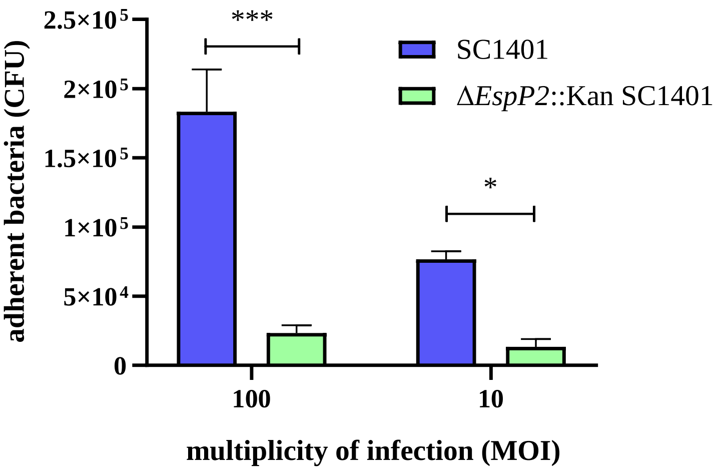

Figure S1. Adhesion of wild type *G. parasuis* and Δ*EspP2*::Kan to NPTr cells. Error bars represent the standard deviation of three independent experiments. Significant differences between groups are indicated by * $p < 0.1$ and *** $p < 0.001$.

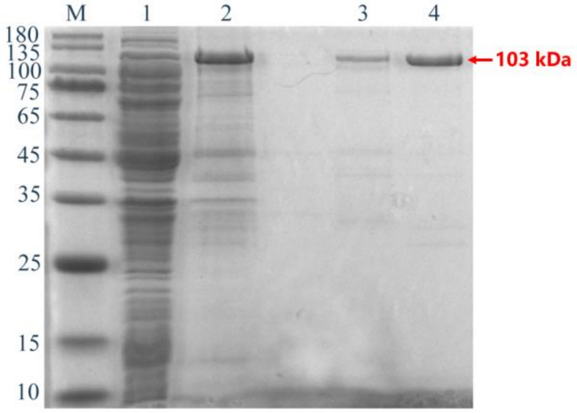

Figure S2. SDS-PAGE analysis of rEspP2. M: Protein molecular standard, 1: pET-32a-rEspP2-BL21 supernatant after ultrasonic crushing, 2: pET-32a-rEspP2-BL21 inclusion body after ultrasonic crushing, 3: purified rEspP2 protein, 4: concentrated rEspP2 protein.

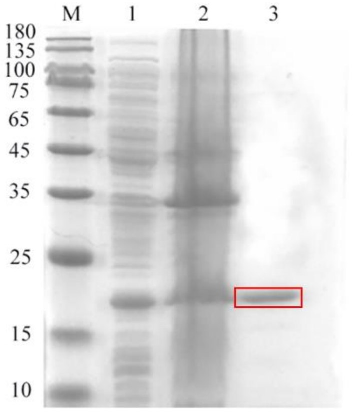

Figure S3. SDS-PAGE analysis of His-tag protein. M: Protein molecular standard, 1: pET-32a-BL21 supernatant after ultrasonic crushing, 2: pET-32a-BL21 inclusion body after ultrasonic crushing, 3: purified His-tag protein.

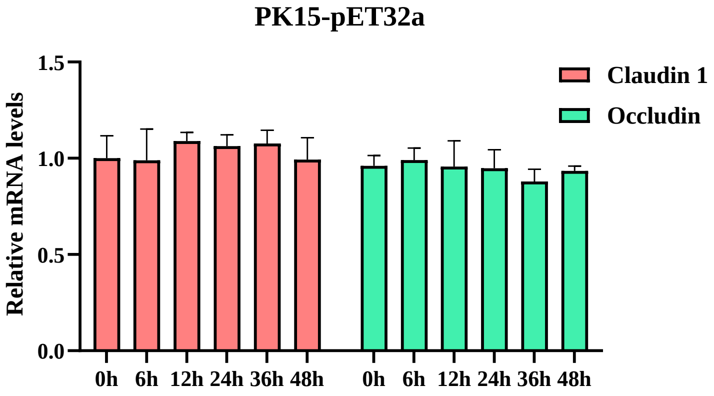

Figure S4. RT-PCR results of *claudin-1* and *occludin* levels in His-tag protein treated PK15 cells.

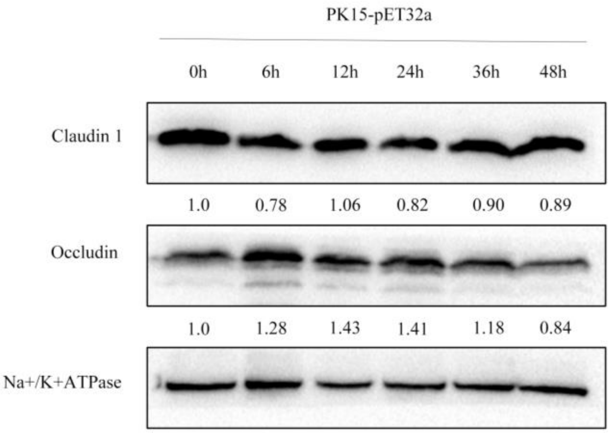

Figure S5. Representative western blot of claudin-1 and occludin in His-tag protein treated PK15 cells. Expression level of claudin-1 and occludin in PK15 cells after incubation with His-tag protein.

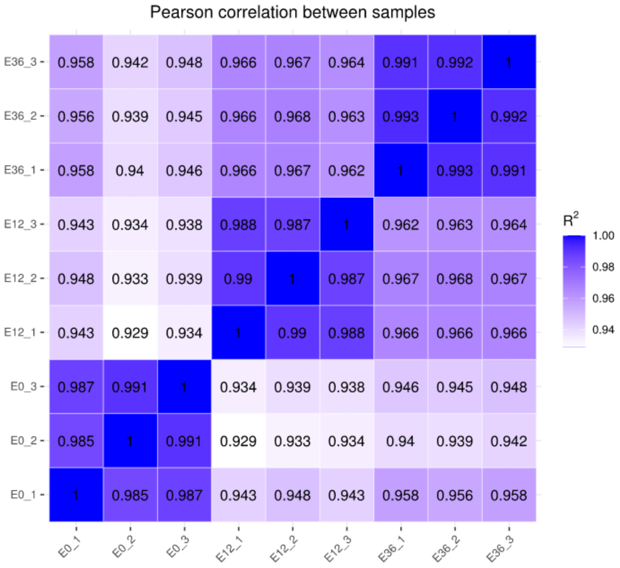

Figure S6. Heat map of the correlation coefficient between samples. E0_1-E0_3 represents three biological duplications of 0 h, E12_1-E12_3 represents three biological duplications of 12 h, E36_1-E36_3 represents three biological duplications of 36 h, R^2 represents the square of Pearson correlation coefficient. R^2 ＞0.95 between the three biological replicates, indicating a good correlation.

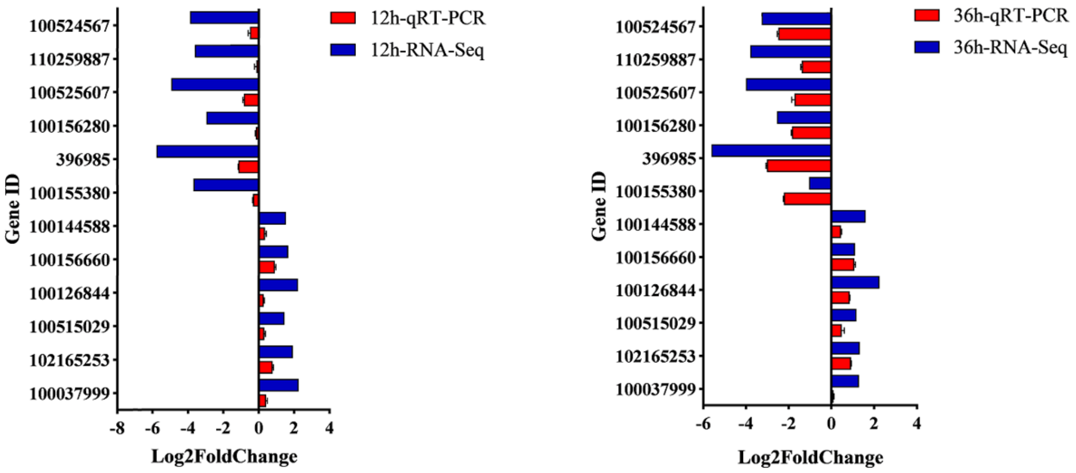

Figure S7. Validation of RNA-Sequencing results by quantitative RT-PCR. Log2 fold change comparison of RNA-Seq (blue bars) and quantitative real-time PCR (red bars) for six differentially expressed genes upregulated at 12 h and 36 h, six genes downregulated at 12 h and 36 h. RT-PCR results showed general consistency with RNA-seq data.
